# Zebrafish (*Danio rerio*) as a Model System to Investigate the Role of the Innate Immune Response in Human Infectious Diseases

**DOI:** 10.3390/ijms252212008

**Published:** 2024-11-08

**Authors:** Maria Franza, Romualdo Varricchio, Giulia Alloisio, Giovanna De Simone, Stefano Di Bella, Paolo Ascenzi, Alessandra di Masi

**Affiliations:** 1 Department of Sciences, Roma Tre University, 00146 Roma, Italy; maria.franza@uniroma3.it (M.F.); romualdo.varricchio@uniroma3.it (R.V.); giulia.alloisio@uniroma3.it (G.A.); giovanna.desimone@uniroma3.it (G.D.S.); paolo.ascenzi@uniroma3.it (P.A.); 2 Clinical Department of Medical, Surgical and Health Sciences, Trieste University, 34127 Trieste, Italy; stefano932@gmail.com; 3 Accademia Nazionale dei Lincei, 00165 Roma, Italy; 4 Centro Linceo Interdisciplinare “Beniamino Segre”, Accademia Nazionale dei Lincei, 00165 Roma, Italy

**Keywords:** animal model, bacteria, fungi, host-pathogen interaction, innate immune response, infectious disease, virus, zebrafish

## Abstract

The zebrafish (*Danio rerio*) has emerged as a valuable model for studying host-pathogen interactions due to its unique combination of characteristics. These include extensive sequence and functional conservation with the human genome, optical transparency in larvae that allows for high-resolution visualization of host cell-microbe interactions, a fully sequenced and annotated genome, advanced forward and reverse genetic tools, and suitability for chemical screening studies. Despite anatomical differences with humans, the zebrafish model has proven instrumental in investigating immune responses and human infectious diseases. Notably, zebrafish larvae rely exclusively on innate immune responses during the early stages of development, as the adaptive immune system becomes fully functional only after 4–6 weeks post-fertilization. This window provides a unique opportunity to isolate and examine infection and inflammation mechanisms driven by the innate immune response without the confounding effects of adaptive immunity. In this review, we highlight the strengths and limitations of using zebrafish as a powerful vertebrate model to study innate immune responses in infectious diseases. We will particularly focus on host-pathogen interactions in human infections caused by various bacteria (*Clostridioides difficile*, *Staphylococcus aureus*, and *Pseudomonas aeruginosa*), viruses (herpes simplex virus 1, SARS-CoV-2), and fungi (*Aspergillus fumigatus* and *Candida albicans*).

## 1. Introduction

The primary organisms used in biomedical research include small models like yeast, *Caenorhabditis elegans*, *Drosophila melanogaster*, *Danio rerio* (commonly known as zebrafish), and large mammalian models, such as mice, rats, and non-human primates. Smaller model organisms offer distinct advantages, including small size, cost-effectiveness, ease of use, and well-characterized biological properties. These attributes make them essential tools for studying disease mechanisms, immune responses, and potential therapeutic interventions in humans [1,2]. Indeed, research involving these small models provide invaluable insights that are often unattainable in human studies due to practical and/or ethical constraints of clinical experiments [1,2].

The selection of animal models for studying a particular human pathogen is influenced by several factors, including susceptibility to infection, physiological similarity to humans, reproducibility, ease of handling, safety, and cost. However, as no model organism can perfectly replicate the human response to infection, different animal models are often used in complementary ways to address specific questions about pathogen-induced diseases. For instance, non-human primate species (NHPs) (i.e., simians—monkeys and apes—and prosimians, such as lemurs) are frequently employed for studying viral pathogens (e.g., bovine spongiform encephalopathy, avian influenza, coronavirus infection) due to their physiological and evolutionary similarities to humans. Moreover, NHPs are often used as models to study viral vaccine efficacy as well as antiviral therapeutic safety and efficacy [3,4,5]. Nonetheless, the use of NHPs is limited by high costs, limited availability, long reproductive cycles, and challenges in genetic manipulation, prompting the use of alternative model systems for studying pathogens. Among other, the laboratory mouse remains one of the most widely used model in infectious disease research. The popularity of mice as a model system is due to several advantages, such as their relatively low cost, ease of housing, rapid reproductive cycles, and large litter sizes. Indeed, the availability of genetically defined inbred mouse strains, humanized mice, and genetically engineered mice lacking specific host genes has significantly advanced infectious disease research, enabling researchers to investigate how pathogens cause diseases, determine the role of specific host genes in disease progression or resistance, and identify potential targets for preventing or treating various infectious agents. Moreover, mouse models deepen our understanding of the immune system’s role in combating infections or mediating vaccine-induced immunity [6]. However, due to the substantial space, labor, and resources required to conduct traditional gene targeting screens, such approaches can be prohibitive.

Over the past two decades, zebrafish have emerged as a valuable model organism for studying embryogenesis, the evolution and development of the immune system, as well as immune-related diseases. Indeed, because of the clear temporal separation between innate and adaptive immune responses, zebrafish represents an excellent model for studying host-pathogen interactions [7,8,9,10,11,12,13,14,15]. Such an advantage is lacking in placentals in which in the first weeks of life the antibodies present in the fetus are of maternal origin. Despite anatomical differences between zebrafish and humans, zebrafish can be used to investigate human infection by injecting a corresponding site that best suits the research question. As a consequence, numerous bacterial and viral infection models have been established in zebrafish to study host-pathogen interactions, chemotactic responses, and inflammation processes in vivo [7,8,16,17,18,19,20,21], providing significant advances in understanding microorganisms pathogenesis and vertebrate host defense mechanisms [13].

This review provides an overview of the benefits and limitations of using zebrafish as a powerful vertebrate model for studying the innate immune response in infectious diseases. Specifically, we provide a description of zebrafish hematopoiesis and the innate immune response, focusing on host-pathogen interactions in various human bacterial, viral, and fungal infections.

## 2. The Zebrafish Model for the Study of Human Infectious Diseases: Advantages and Disadvantages

*D. rerio* is a small freshwater fish native to the tropical waters of South Asia, belonging to the phylum *Chordata*, infraphylum Gnathostomata, class *Actinopterygii*, infraclass Teleostei, order *Cypriniformes*, and family *Cyprinidae* [22]. Gnathostomes (i.e., jawed vertebrate) are divided into two major groups: (i) cartilaginous fish, such as sharks, rays, and chimaeras, and (ii) bony vertebrates (Osteichthyes), which include both zebrafish and mammals. The evolutionary paths of zebrafish and mammals within the bony vertebrates diverged approximately 400–450 million years ago [22]. As part of the gnathostome lineage, zebrafish possess a jaw apparatus, enabling them to exploit new food sources and contributing to their evolutionary success. Additionally, zebrafish have evolved pectoral and pelvic fins, which are crucial adaptations for locomotion [23,24].

The widespread adoption of zebrafish as a model organism is largely credited to George Streisinger, who in the 1980s identified this small fish as a promising model for studying forward genetics [25,26,27]. Zebrafish have been used for almost 30 years as a model to study developmental biology and hematopoiesis because larvae are optically accessible and develop rapidly [28,29]. However, it is only in the last two decades that the interest of the scientific community in zebrafish has expanded to include studies on human diseases, cancer, and immunology, previously investigated mainly using murine models. This shift has been facilitated by two key factors: (i) the annotation of the zebrafish genome (http://www.sanger.ac.uk/Projects/D_rerio/, accessed on 3 August 2024), which enables the creation of mutant zebrafish lines to investigate gene function; and (ii) the genetic and physiological similarities between zebrafish and humans, with approximately 70% of human genes having a counterpart in zebrafish [8,10,14,30]. Advances in zebrafish genome sequencing and gene editing technologies have enabled the development of transgenic fish used in different research areas including immunotoxicity [31,32,33] as well as toxicological and ecotoxicological studies [34,35,36].

The zebrafish model adheres to the principle of the 3Rs (Replacement, Reduction, and Refinement), as required by numerous national and international regulatory entities [21]. Indeed, the use of zebrafish in research reduces the time and resources needed compared to other animal models, while providing more informative and predictive results than those obtained from in vitro studies [21,37]. Consequently, zebrafish models allow for the replacement and reduction of mammalian models in research, thereby mitigating concerns related to the welfare of these animals [21,37].

Zebrafish offers numerous advantages that complement mammalian models, including low costs, small size (less than 5 cm), high reproductive rate (200–300 new progeny per week), relatively rapid life cycle, and ease of breeding [21,28,38,39,40,41,42,43,44,45,46]. Additionally, zebrafish develop ex utero, and the transparency of their embryos allows for the in vivo study of ontogeny from the earliest stages of development [14,30]. Numerous transgenic zebrafish lines have been developed with fluorescent markers in various immune cells (e.g., neutrophils, macrophages, T cells), facilitating the visualization of host-microbe interactions in transparent larvae and the study of inflammatory processes relevant to human health. Fluorescent cells can be tracked in real-time in live transgenic fish using fluorochromes to tag cells or with whole-mount in situ hybridization (WISH) in fixed embryos [8]. For example, to characterize the immune response to pollutants, biomarkers such as neutrophil activation and migration as well as macrophage migration can be monitored in real-time using specific transgenic lines like *Tg*(*mpx: GFP*), *Tg*(*lyz: DsRed 2*), and *Tg*(*lyz: EGFP*) lines [18,36] or fluorescent T cells [41]. These biomarkers, combined with other molecular techniques such as the analysis of genes involved in macrophage migration and adaptive immunity markers, have allowed a deeper evaluation of the inflammatory response, especially after fish exposure to micro and nanoparticulate materials [36,47,48,49,50,51,52].

Stress may pose a serious challenge to immune homeostasis in zebrafish [53]. The implementation of fish welfare protocols is crucial for reducing overall stress levels, which in turn enhances the success of experiments and minimizes procedural errors arising from undesirable behaviors [54,55,56,57]. To achieve this, it is important to establish a consistent feeding schedule that includes a balanced diet of commercial food, supplemented with live or frozen arthropods. Creating a “stress-free” environment also involves minimizing lighting, noise, and vibrations around the aquarium, as well as avoiding overcrowding and sudden changes in water quality [56]. Non-invasive observation and regular monitoring of fish behavior are essential for detecting signs of stress, enabling timely interventions to ensure their well-being. The characteristics of the environment significantly influence fish health, making it important to maintain optimal conditions in the aquarium, such as a temperature range of 24 to 28 °C, a neutral pH (6.5–7.5), and adequate water oxygenation [56,58]. The presence of both hiding spots and open areas in the tank is essential for reducing stress, as zebrafish are social animals that benefit from an environment resembling their natural habitat [47,48,49,50].

## 3. Hematopoiesis and Innate Immune Response in Zebrafish

Despite some differences that relate to the location and timing of immune cell development, many hematopoiesis pathways and regulatory processes underlying immune cell development are highly conserved between zebrafish and humans [15,40,59,60].

The zebrafish innate immune system is functional during the embryonic stage, whereas the maturation and functionality of the adaptive immune system occur around 3 to 6 weeks post-fertilization (wpf), when lymphocytes become functional [9,15,16,28,41,42,61,62,63,64,65]. Both the innate and adaptive immune system play a key role in the maintenance and repair of tissues during health and disease [66]. The innate immune system serves as the first line of defense against infectious agents, including bacteria, viruses, fungi, and parasites [43,45]. Unlike the adaptive immune system, which tailors its response to specific pathogens over time, the innate immune system provides an immediate but non-specific response. This system comprises various components, including physical barriers (such as skin and mucous membranes), cellular defenses (e.g., neutrophils and macrophages), and molecular mechanisms (e.g., pattern recognition receptors (PRRs), cytokines, inflammasome) [67,68]. Together, these elements recognize and initiate an inflammatory response to eliminate pathogens and prevent infection [43,69]. In zebrafish, kidneys function as the primary lymphoid organ, similarly to the mammalian bone marrow, and contain various hematopoietic cell types such as macrophages, neutrophils, and lymphocytes [40,70,71].

The high efficiency of performing large-scale infection and chemical treatments in zebrafish facilitates the identification of novel microbial virulence factors and enables high-throughput compound screening to study disease mechanisms at the cellular level. Chemicals can be easily added to the water, and molecule screenings, especially those involving fluorochromes, can be conducted on a large-scale using embryos arranged in 96-well plates [8,9,14,41,72]. Additionally, in zebrafish larvae, rapid systemic infection can be initiated by direct microinjection of bacterial suspension into the bloodstream. For more localized infections, microbes can be injected into the muscle tail or hindbrain ventricle. To achieve high transfer rates, microbes can be injected into the yolk within the first few hours after fertilization [21,73]. The absence of immune cells in the yolk is crucial, as it allows bacteria to proliferate freely before invading the larval tissues [21,47].

Despite the numerous advantages, the use of zebrafish model to study innate immunity requires validating the findings in other model systems, such as mammals [21,41,66]. Indeed, zebrafish do not exhibit the rapid and robust adaptive immune responses that characterize mammals. While organized lymphoid tissues are present in other fish and lower vertebrates, zebrafish possess a unique iteration of the immune system that includes the cellular components of the adaptive immune system but lacks the structures that facilitate antigen presentation and intricate interactions between immune cells [8,21,74]. Moreover, as mammals and fish live in distinct environments, they have different physiology, gene expression, and gene regulation. This can result in significant differences in their susceptibility to certain pathogens, thereby limiting the relevance of infection models for some human-specific diseases [14,75].

### 3.1. Hematopoiesis in Zebrafish

**Figure 1:**
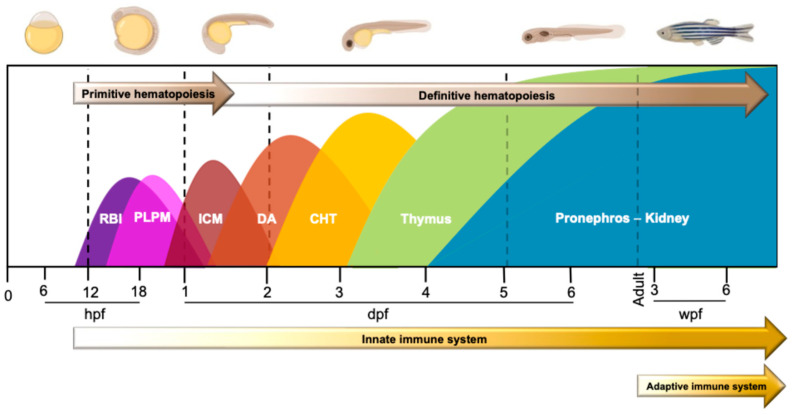
Hematopoiesis and development of the immune system in zebrafish. The development of the immune system starts with primitive hematopoiesis at 11 h post-fertilization (hpf). Myeloid and erythroid cells originate in the anterior lateral plate mesoderm (ALPM) and posterior lateral mesoderm (PLPM)). Specifically, myeloid cells develop in the rostral blood islands (RBI) and erythroid cells in the intermediate cell mass (ICM), respectively. At about 2 days post-fertilization (dpf), hematopoietic stem cells (HSCs) appear in the dorsal aorta (DA) and then transit into the caudal hematopoietic tissues (CHT). The terminal phase of hematopoiesis involves the migration of HSCs to the thymus and pronephros (i.e., the first stage of kidney development), where the full maturation of the blood cells occurs. Notably, at 3 dpf zebrafish emerge from the chorion and take contact with the outside environment without fully developed CD4^+^/CD8^+^ lymphocytes, which appear at 3 weeks post-fertilization (wpf).

Hematopoiesis is a complex process involving a multitude of signaling pathways that influence each stage of blood cell differentiation, from the earliest precursors to the final state of maturation [76]. In mammals, the organization of the immune system into innate and adaptive components is based on hematopoiesis from distinct blood precursors that undergo an endothelial-hematopoietic transition mechanism during development [60,77]. Despite anatomical differences, the genetic and regulatory networks of hematopoiesis in zebrafish are similar to those described in humans; in fact, the molecular mechanisms underlying this process are highly conserved between the two species [40]. In zebrafish, hematopoiesis process occurs in two sequential events: (i) the primitive hematopoiesis that takes place in the early embryonic development and gives rise to erythroid and myeloid progenitors; and (ii) the definitive hematopoiesis, which gives rise to hematopoietic stem and progenitor cells (HSPCs) that are responsible for generating all adult blood cells [29,60,78,79] (Figure 1).

Primitive hematopoiesis begins at around 11 h post-fertilization (hpf) in two distinct anatomical areas (i.e., anterior lateral plate mesoderm (ALPM) and posterior lateral mesoderm (PLPM)) and generates erythrocytes, which support tissue oxygenation during the rapid growth of the embryo, as well as macrophages, which engulf pathogens and clear apoptotic cells produced naturally during development [60,61,80]. In detail, myeloid progenitor cells (MCPs) originate in the rostral blood islands (RBI), which is located in the ALPM, and then migrate to the rostral blood pool (RBP) where they differentiate into macrophages and neutrophilic granulocytes [70,81,82]. Microglia, which differentiates from these primitive macrophages, plays a critical role in regulating neural development and function in the central nervous system [29]. Therefore, even though the cells arising from this initial hematopoietic wave are not pluripotent and transient, defects in primitive hematopoiesis can lead to significant developmental consequences.

At 1 day post fertilization (dpf), a second transient hematopoietic event occurs in the intermediate cell mass (ICM) blood islands, which develop at the trunk midline from two bilateral stripes of PLPM. The ICM is analogous to the extra-embryonic yolk sac blood islands of mammals [8,81,83,84,85] (Figure 1).

Definitive hematopoiesis starts at around 30 hpf (~1.5 dpf) and continues until zebrafish adulthood, producing hematopoietic stem cells (HSCs), which are multipotent cells that can generate all blood lineages in the adult organism, including lymphoid lineages [86]. The processes leading to the generation of HSCs in fish and mammals are similar [60,77,87]. HSCs arise from hemogenic endothelial cells, lining the ventral wall of the dorsal aorta (DA), corresponding to the aorta-gonadal-mesonephro region in mammals [60,77]. Starting from 2 dpf, a subset of HSCs migrates to the caudal hematopoietic tissue (CHT), which is functionally homologous to the fetal mammalian liver and contributes to HSCs growth and differentiation before their final deposition in the kidney, the human bone marrow equivalent [29,88]. Indeed, the terminal phase of hematopoiesis (3–4 dpf) involves the migration of HSC to the thymus and pronephros (i.e., the first stage of kidney development) [89,90,91,92], the sites where occur the full functional maturation of the blood lines necessary for the physiological functioning of the adult zebrafish immune system [16,93]. Despite the absence of lymph nodes in zebrafish, evidence suggests that the lymphatic system begins to develop between 3 and 5 dpf [94,95]. The spleen- and gut-associated lymphoid tissue (GALT) are analogous in function to humans; these organs are critical for immune responses and the maintenance of immune homeostasis [96] (Figure 1).

Notably, the adaptative immune system is activated following the maturation of (i) CD4^+^/CD8^+^ T lymphocytes, which appear at 3 weeks post fertilization (wpf) [9,15]; and (ii) B cells, which develop in the kidney marrow and are responsible for the production of antibodies involved in humoral immunity [15,62,97]. Unlike mammals, which produce several classes of immunoglobulins (IgM, IgG, IgA, IgE, and IgD), zebrafish primarily produce IgM, IgD and IgZ/T [97,98,99]. IgM/IgD are the first antibody produced during an infection and participates in the primary immune response, whereas IgZ/T, a unique class absent in mammals, is thought to be involved in the mucosal immunity [28,98].

### 3.2. The Innate Immune Response in Zebrafish

#### 3.2.1. The Innate Cellular Components

**Figure 2:**
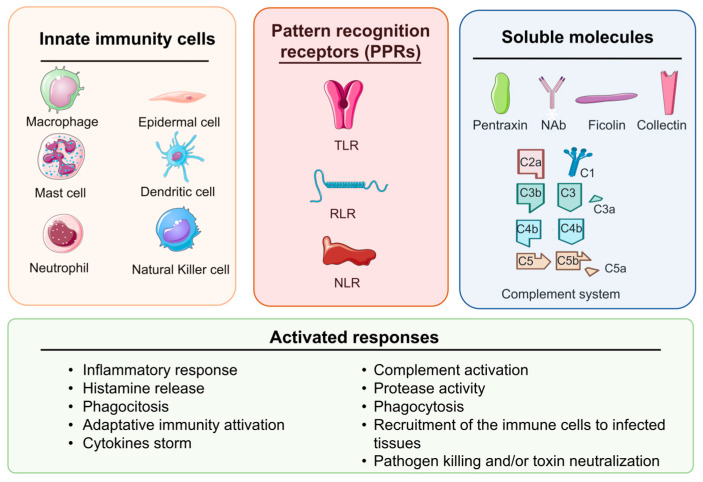
The innate immune response in zebrafish. The innate immune system is a complex composition of cellular and humoral components. The figure shows the immunity cells, the pattern recognition receptors, and the soluble components that coordinate the diverse innate immunity responses. The figure has been partially generated using the website Servier Medical Art, provided by Servier, licensed under a Creative Commons Attribution 3.0 unported license.

Zebrafish possess innate cellular components that are structurally and functionally similar to those of mammals, playing a key role in phagocytosis, pathogen clearance, and inflammation capabilities [100,101]. Several cell types of the myeloid lineage (i.e., dendritic cells, epidermal cells, macrophages, mast cells, and neutrophils) are responsible for the detection and clearance of infectious microorganisms [102]. In addition to the various myeloid cells, the natural killer (NK) cells, derived from lymphoid precursors, are also considered part of the cellular innate immune system [15,103] (Figure 2).

Macrophages serve as phagocytic cells responsible for ingesting and breaking down pathogens and debris, playing a vital role in inflammation and tissue remodeling [104,105,106]. In zebrafish, primitive macrophages first appear during the early 13-somite stage (15 hpf) of embryo development [16,61]. After differentiating in the yolk sac, they acquire the ability to engulf dead cells and clear bacteria from circulation [61]. From the yolk sac, some macrophages migrate into epithelial tissues [107,108,109,110,111], while others enter the bloodstream [16,61].

Neutrophils, a type of white blood cell, serve as the body’s first line of defense, responding to systemic inflammatory signals to help restore homeostasis [102,112,113]. As zebrafish mature from the larval stage to adulthood, neutrophils experience changes in the structure of their nuclear envelope [114], which enhances their adaptability and ability to migrate to tissues where inflammation occurs. To reach infection sites, neutrophils mobilize from haemopoietic tissues via the vasculature. Upon reaching the site of infection, neutrophils initiate several key immune responses including phagocytosis of microbes, secretion of granular proteins and antimicrobial substances, production of reactive oxygen species (ROS), and the release of neutrophil extracellular traps (NETs) [115,116]. These actions play a crucial role in the early stages of immune defense [115,116].

Functionally, both neutrophils and macrophages express PRRs on their surface [102,117], which detect pathogen-associated molecular patterns (PAMPs) and damage-associated molecular patterns (DAMPs), thus triggering inflammatory cascades including the activation of transcription factors like nuclear factor kappa-light-chain-enhancer of activated B cells (NFκB), interferon (IFN) regulatory factors (IRFs), and activator protein-1 (AP-1), which in turn promote the expression of pro-inflammatory cytokines, chemokines (CC), and IFNs [69,101,102].

#### 3.2.2. The Innate Immune Response Mediators

1. Pattern recognition receptors

Inflammatory responses are triggered by the activation and signaling of three main classes of PRRs: Toll-like receptors (TLRs), RIG-I-like receptors (RLRs), and NOD-like receptors (NLRs) [41,118,119,120,121,122].

Among these, TLRs are the most extensively studied, as their activation forms a critical link between the innate and adaptive immune systems [123]. Genomic analyses have identified approximately 24 TLR receptor variants in zebrafish, two of which are unique to fish (TLR21 and TLR22), and 10 of which are common to humans [12]. Additionally, zebrafish possess homologues for genes encoding key TLR adaptor proteins, such as myeloid differentiation primary response 88 (MyD88), TIR-domain-containing adapter-inducing IFN-β (TRIF) and sterile alpha and TIR motif-containing protein 1 (SARM1) [124,125,126].

Zebrafish possess orthologs of several NLRs, including: (i) the nucleotide-binding oligomerization domain-like receptor 1 (NOD1) and 2 (NOD2), and (ii) the NLR family pyrin domain containing 1 (NLRP1) and 3 (NLRP3). However, zebrafish also express more than 400 unique NLR genes [122]. These NLRs are localized in the cytosol of macrophages and are involved not only in inflammasome formation but also in the activation of the NF-κB and mitogen-activated protein kinase (MAPK) pathways [127], leading to the generation of pro-inflammatory signals. In contrast, RLRs and their associated signaling pathways are highly conserved in zebrafish. The species has orthologs of key RLRs, including retinoic acid-inducible gene I (RIG-I), melanoma differentiation-associated protein 5 (MDA5), and laboratory of genetics and physiology 2 (LGP2) [128,129].

2. The soluble molecules

Several molecules present in extracellular fluids play a vital role in innate immunity by detecting and neutralizing pathogens. Together, these soluble effectors form the humoral branch of innate immunity and include natural antibodies (NAbs), pentraxins, collectins, ficolins, and the complement system. These molecules function through three primary mechanisms: (i) acting as opsonins to enhance phagocytosis; (ii) initiating inflammatory responses to recruit immune cells to infected tissues; and (iii) directly killing pathogens or neutralizing their toxins [130,131] (Figure 2).

Cytokines, a family of small secreted proteins including lymphotoxins, interleukins (ILs), CCs, and IFNs regulate various stages of inflammation [132]. The most significant pro-inflammatory cytokines are tumor necrosis factor alpha (TNFα) and interleukin-1 beta (IL1β). IL1β plays a key role in initiating inflammation through inflammasome formation. In zebrafish embryos and larvae, IL1β is induced in response to injury and infections, similar to humans, facilitating the recruitment of white blood cells to the inflammation site and modulating myelopoiesis through the NF-κB and CCAAT/enhancer-binding protein beta (C/EBPβ) signaling [133,134,135] (Figure 2). Also CC, which are a subgroup of cytokines with chemotactic properties, are pivotal in directing leukocytes to sites of inflammation [136]. In zebrafish, over 80 putative genes belonging to the CC subclass have been identified [137,138,139].

IFNs are small, secreted proteins that play a critical role in immune responses. In vertebrates, they are divided into four classes [140]. IFNs I, III, and IV are key components of innate immunity, crucial for the clearance of viral infections, while IFN II are immunomodulatory and essential for controlling intracellular bacterial pathogens [141]. Functionally, zebrafish IFN are similar to human IFN, as they activate the transcription of a wide range of IFN-stimulated genes (ISGs) that mediate inflammation and serve as antiviral defenses by binding to their cytokine-like receptors [142,143,144,145,146].

Inflammasomes are key components in the inflammatory response, consisting of large complexes formed by multiple copies of three distinct proteins. These include: (i) a receptor that detects PAMPs or DAMPs and that belongs to NLR, RLR, or ALR (AIM2-like receptors) family; (ii) a pro-caspase, typically pro-caspase-1, which becomes activated through oligomerization after being recruited into the inflammasome; and (iii) an adaptor protein, namely ASC (apoptosis-associated speck-like protein containing a caspase recruitment domain or CARD), which puts in connection the activated receptor with the pro-caspase [147]. The primary function of inflammasomes is to activate the cysteine protease caspase-1, which processes pro-interleukins IL1β and IL18 into their active forms, thereby promoting inflammation and triggering pyroptosis, a highly inflammatory form of programmed cell death. Caspase-1-dependent inflammasomes are referred to as canonical; however, non-canonical inflammasomes have also been identified in mammals, which activate other caspases such as CASP4, CASP5, CASP8, and CASP11 [148,149]. In the zebrafish genome, four CASP1-like genes have been identified, two of which (i.e., caspA and caspB), are associated with inflammasome [150,151].

## 4. Zebrafish as a Model to Investigate the Role of Innate Immune Response in Human Infectious Diseases

Several infection strategies, including immersion, microinjection, and microgavage, have been employed to induce systemic or local infections in zebrafish with different microorganisms [152,153]. Each method has its own advantages and disadvantages, and the choice of strategy depends on the intended route of infection.

The immersion method entails exposing fish or larvae to a liquid medium, typically water, that contains the pathogen or the pathogenic molecules (e.g., toxins). However, this technique poses challenges in accurately monitoring or controlling the route and timing of administration, which increases the risk of unintended toxicity and off-target effects [154]. In contrast, microinjection is a technique that directly introduces pathogens or specific molecules into target tissues or body cavities of zebrafish larvae or embryos. This method is well-suited for investigating host-pathogen interactions, immune responses, and tissue-specific effects. Both microinjection and microgavage enable more controlled and consistent administration of materials into the body, effectively addressing some of the limitations associated with the immersion strategy [155,156]. Likewise, the microgavage method is employed to deliver pathogens or chemicals such as drugs directly into the gastrointestinal tract, specifically the anterior intestine, of larval zebrafish. These methods are designed to overcome the limitations of immersion exposure, providing more controlled, consistent delivery of materials into the body [155,156].

**Figure 3:**
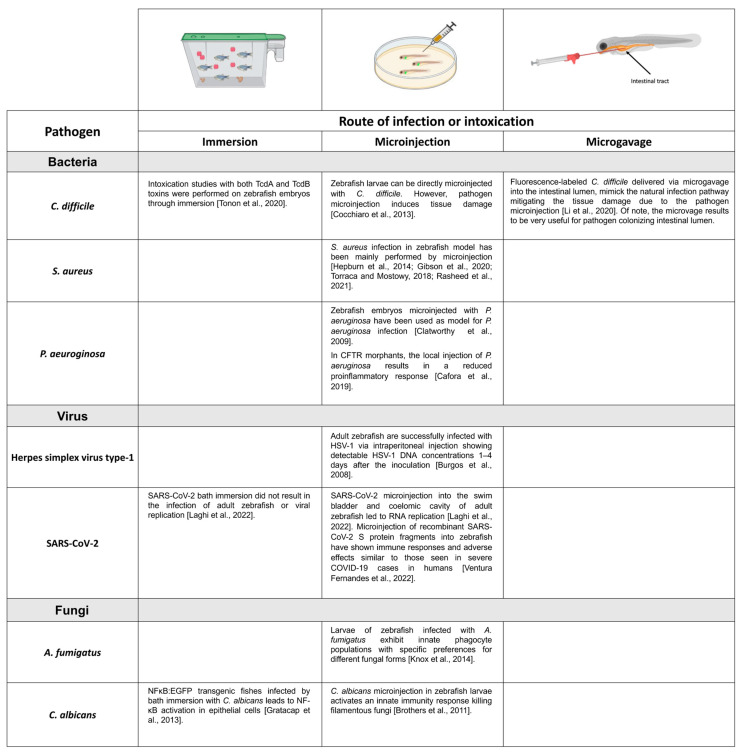
Summary of the infection strategies (i.e., immersion, microinjection, and microgavage) used to induce systemic or local infections/intoxication in zebrafish with bacteria, viruses, and fungi [152,157,158,159,160,161,162,163,164,165,166,167,168,169,170].

In this section, we will provide examples of how the zebrafish model is used to study innate immune responses to human infections caused by various pathogens, including bacteria (*Clostridioides difficile*, *Staphylococcus aureus*, and *Pseudomonas aeruginosa*), viruses (herpes simplex virus type 1, and SARS-CoV-2), and fungi (*Candida albicans* and *Aspergillus fumigatus*) (Figure 3).

### 4.1. Zebrafish Model to Study Human Infectious Diseases Caused by Bacteria

#### 4.1.1. *Clostridioides difficile*

*C. difficile* is an anaerobic, Gram-positive, spore-forming, toxin-producing bacterium and a leading cause of hospital-acquired infections in Western countries. Clinical manifestations of *C. difficile* infection (CDI) can vary from asymptomatic colonization and mild diarrhea to severe complications, such as toxic megacolon and life-threatening colitis. The pathogenicity of *C. difficile* primarily arises from its toxins A (TcdA) and B (TcdB), which enter host cells via receptor-mediated endocytosis, leading to cytotoxic effects [157,171,172,173,174,175].

Zebrafish has been used to evaluate the immune response to CDI [158,176]. Methods used to induce CDI in zebrafish larvae include microinjection and microgavage [73,159]. Similarly to mice [177], *C. difficile* was detected in the intestines of zebrafish at 24 h post-infection (hpi) only in gnotobiotic fish (i.e., animals in which normal host microbiota has been replaced by a defined set of microbes) or in animals pretreated with antibiotics [158]. Conversely, at later timepoints (i.e., 48, 72, and 120 hpi), *C. difficile* grew only in media containing taurocholic acid (TCA), which stimulates *C. difficile* spore germination [158]. Of note, antibiotic-treated zebrafish did not exhibit symptoms of CDI (e.g., intestinal neutrophil influx or death) despite the reported *C. difficile* proliferation. This may be explained by the fact that the injection can induce tissue damage [159], thus activating innate immune cells (i.e., macrophages and neutrophils) that quickly detect *C. difficile* at the site of injection, preventing its spread throughout the organism. Moreover, the structural differences between zebrafish and mammalian intestines, such as the lack of intestinal crypts and the different maintenance temperatures, possibly contribute to the absence of a CDI phenotype shortly after infection [158]. To avoid tissue damage, microgavage protocols have been set up to directly deliver *C. difficile* into the intestinal lumen of zebrafish, mimicking the natural route of infection [158,176,178,179]. Results showed that both neutrophils and macrophages can recognize *C. difficile* in infected zebrafish, but they only migrate to the gastrointestinal tract 12 h post-microgavage [158].

Zebrafish has been also used to study the mechanism of action of *C. difficile* toxins. Intoxication studies were performed by immersion of embryos in water containing tolerated doses of either TcdA or TcdB or both toxins. Results obtained indicated that the intoxication induces neutrophils recruitment, which in turn promote pro-inflammatory cytokines production (e.g., IL1β, IL6, and IL8) [157]. Moreover, neutrophils promote angiogenesis and induce vascular permeability through the vascular-endothelial growth factor (VEGF) production [157,180,181].

Interestingly, zebrafish models of CDI have shed light on the role of novel components in the innate immune response. Over the last 15 years, numerous clinical studies have shown a significant association between low levels of human serum albumin (HSA) and the development of CDI, suggesting that hypoalbuminemia predisposes patients to severe/recurrent episodes [182,183,184,185]. The molecular mechanism underlying this phenomenon is linked to the HSA ability to bind and neutralize TcdA and TcdB, thus preventing host cell damage [157,175,186]. The use of a zebrafish embryo model further supports these findings, as demonstrated by increased survival rates of embryos exposed to *C. difficile* toxins in the presence of HSA, compared to those treated only with TcdA or TcdB [157,186]. Notably, HSA also protected zebrafish embryos from toxin-induced inflammatory responses [157]. These findings suggest that HSA acts as a serum buffer, partially neutralizing toxins that enter the bloodstream, contributing to the innate immune response. In hypoalbuminemic individuals, this buffering activity is impaired, reducing the effectiveness of toxin neutralization and potentially leading to more severe clinical outcomes in CDI [157,175,186].

#### 4.1.2. *Staphylococcus aureus*

*S. aureus* is a Gram-positive bacterium and the major cause of clinical infections in humans, leading to conditions such as skin and soft tissue infections, pneumonia, bloodstream infections, endocarditis, and septic arthritis. Specific syndromes can also arise due to the local or systemic effects of certain toxins [187]. The increasing antibiotic resistance of this pathogen, along with its prevalence in clinical infections, poses a significant threat to human health. While *S. aureus* is not a natural pathogen for zebrafish, it exhibits acute infection symptoms in both embryos [188,189,190] and adults [160,191].

Neutrophils serve as the primary niche for bacterial replication and clone selection, and their depletion can significantly reduce this process [161]. In *S. aureus* infection, the p62-mediated autophagy and Lc3-associated phagocytosis (LAP) by neutrophils have opposing roles [190]. Specifically, p62-selective autophagy promotes bacterial clearance, while LAP facilitates the establishment of an intracellular niche for the bacteria. Notably, the loss of p62 activity is sufficient to increase mortality following *S. aureus* infection. Additionally, p62 knockdown using morpholinos significantly heightened susceptibility to *S. aureus* infection. This represents the first in vivo evidence highlighting the importance of p62 in intracellular *S. aureus* damage [161].

*S. aureus* infection in zebrafish model has been mainly performed by microinjection [152,160,161,162]. The zebrafish model has been crucial in elucidating the role of nerve growth factor beta (NGF-β) in innate immunity during *S. aureus* infection [162]. The activation of NLRs through the recognition of *S. aureus* exoproteins stimulates macrophages to release NGF-β, which subsequently enhances bacterial killing. Notably, mutations in NGF-β or in its high-affinity receptor, the tropomyosin receptor kinase A (TrkA), are associated with increased severity of *S. aureus* infections in humans. Zebrafish lacking the orthologous TrkA also exhibit heightened susceptibility to *S. aureus* infection, supporting the notion of an evolutionarily conserved role for the NGF-β-TrkA axis in host defense [152,162].

In systemic infections of zebrafish with *S. aureus*, macrophages and neutrophils eliminate most of the injected bacteria. However, some persistent colonies evade the phagocyte-mediated immune response, allowing them to survive and replicate. This situation creates an immunological bottleneck, resulting in clonal selection [152]. Notably, as *S. aureus* can develop antibiotic resistance, the administration of sub-curative doses of antibiotics in zebrafish may result in the expansion of antibiotic-resistant clones in vivo. This phenomenon is associated with phagocyte-dependent clonal selection, which is a characteristic feature of *S. aureus* infections [152]. Overall, these findings have significant implications for developing novel therapeutic strategies aimed at reducing disease severity and limiting the emergence of antimicrobial-resistant strains.

#### 4.1.3. *Pseudomonas aeruginosa*

*P. aeruginosa* is a leading cause of hospital-acquired infections, particularly in patients who are injured, burned, or immunocompromised. It is also the primary cause of death in individuals with cystic fibrosis (CF). This Gram-negative bacterium is highly adaptable and thrives in diverse environments, including water and soil, often in association with other eukaryotic organisms. Given the similarities between zebrafish and mammalian immune responses, zebrafish embryos have been extensively used as model for *P. aeruginosa* infection [163,192,193,194].

Clatworthy and coworkers (2009) assessed the zebrafish embryonic immune response to *P. aeruginosa* mutants. Thanks to the transparency of zebrafish embryos, researchers were able to visually confirm that *P. aeruginosa*-GFP labeled bacteria were engulfed by both myeloperoxidase-positive neutrophils and macrophages. This allowed to counteract the pathogenesis of the infection [163].

Most individuals with CF, a genetic disorder caused by mutations in the cystic fibrosis transmembrane conductance regulator (CFTR) gene, are colonized by *P. aeruginosa*, which represent the major cause to morbidity and mortality in these patients [194]. Indeed, the impaired function of the CFTR chloride channel in CF patients leads to thick mucus accumulation in the lungs, creating an environment that is particularly conducive to the proliferation of *P. aeruginosa*. Results obtained using the zebrafish CF model, which strongly resembles the phenotype of CF patients (e.g., severe pancreatic dysfunction and anemia) [194,195,196], suggest that the CFTR channel also plays a role in the innate immune response, contributing to the bactericidal activity of macrophages [194,197,198,199]. In CFTR morphants, the production of ROS is significantly reduced compared to control embryos, and a decrease in neutrophil migration toward the injection site is observed following local injection, supporting a link between CFTR and the innate immune response [200]. Indeed, CF zebrafish embryos exhibit a diminished proinflammatory immune response following bacterial infection compared to wild-type embryos, as indicated by significantly lower TNFα and IL1β responses [164,200]. Collectively, these findings suggest that CFTR moderately contributes to resistance against *P. aeruginosa* infection in zebrafish, likely due to alterations in the inflammatory response and potentially related to changes in the bactericidal function of innate immune cells [201].

### 4.2. Zebrafish Model to Study Human Infectious Diseases Caused by Viruses

Defense against viral infections depends on both the innate and adaptive immune systems. Focused studies of the innate immune response to viral infections can be conducted using the zebrafish model, as there is a 4 to 6-week developmental window during which they possess a functional innate immune system. During viral infections, TLRs play a crucial role [2,202].

#### 4.2.1. Herpes Simplex Virus 1

Herpes simplex virus 1 (HSV-1) is a double-stranded DNA virus that is primarily transmitted through saliva or other body fluids in humans. While commonly associated with cold sores, HSV-1 can also cause severe infections (especially in immunocompromised individuals) such as encephalitis, pneumonia, and hepatitis. Zebrafish serve as a valuable model for studying HSV-1 infection and its effects on the nervous system [165].

Adult zebrafish have been successfully infected with HSV-1 through intraperitoneal injection, with detectable HSV-1 DNA concentrations observed 1 to 4 days post-inoculation [165]. Initially, the infection was localized to the abdominal cavity but ultimately spread to the nervous system, including the brain [165]. Zebrafish has also been used to explore HSV-1 infection dynamics, and especially the innate immune response, across various stages of larval development, ranging from 48 to 96 hpf. Notably, 72 hpf was identified as the optimal stage for inducing HSV-1 infection, which triggered the expression of several antiviral genes (e.g., IFN-1, ISG15, and viperin) involved in the activation of the innate immune responses [203].

HSV-1 infection in zebrafish has also demonstrated that the stimulator of IFN cGAMP interactor (STING) activates the TBK1 kinase and the IRF3 transcription factor, which subsequently induces the expression of a wide range of cytokines and chemokines crucial for modulating the innate immunity [203]. Notably, silencing the STING protein abolished the expression of IFN-1, ISG15, and viperin. This finding highlights the potential for screening compounds that specifically target the STING signaling pathway [203].

#### 4.2.2. SARS-CoV-2

SARS-CoV-2, a member of the family *Coronaviridae* and order *Nidovirales*, is the etiologic agent of COVID-19. The virus has a single-stranded, positive-sense RNA genome and contains key structural proteins: spike (S), membrane (M), envelope (E), and nucleocapsid (N) proteins [204]. Zebrafish have contributed significantly to understand COVID-19 pathogenesis and to study the mechanisms of action of SARS-CoV-2 viral proteins [153,166,202].

Microinjection of recombinant S protein fragments into zebrafish triggered an immune response and adverse effects resembling those seen in severe COVID-19 cases in humans. Moreover, histological analysis revealed liver abnormalities (e.g., lymphocyte infiltration, sinusoidal dilation, necrosis, and steatosis) closely mirroring those observed in COVID-19 patients [167,205]. From a molecular point of view, the zebrafish model of COVID-19 allowed to demonstrate that the hyperinflammation triggered by the S protein requires activation of both the Tlr2/Myd88 (Toll-like receptor 2/Myeloid differentiation primary response 88) and inflammasome signaling pathways, independent of IL1β production [202]. These findings also emphasize the critical role of the inflammasome in S protein-induced emergency myelopoiesis, a process that replenishes innate immune cells in peripheral tissues during inflammation. Interestingly, unlike in humans, emergency myelopoiesis in the zebrafish model occurs independently of the Tlr2/Myd88 signaling pathway [153,202].

Recent studies have investigated the ecotoxicity and physiological effects of SARS-CoV-2 on both zebrafish larvae and adults. It was found that while immersion exposure of larvae to the virus does not result in infection or viral replication, microinjection into the swim bladder and coelomic cavity triggers RNA replication [166]. Additionally, exposing adult zebrafish to SARS-CoV-2 led to behavioral and physiological disruptions, including impaired habituation memory, diminished antipredatory responses, and mutagenic effects. The virus also caused biochemical alterations such as redox imbalance, cholinesterase inhibition, nitrosative stress, and inflammation, which contributed to DNA damage and nuclear abnormalities in erythrocytes. These findings suggest that SARS-CoV-2 can affect neural networks and physiological processes in zebrafish [206].

In COVID-19, a strong correlation has been observed between low HSA levels and increased mortality risk, as well as more severe responses to SARS-CoV-2 [207,208,209]. Previous research has highlighted the importance of HSA levels during COVID-19 due to its protective roles, including its ability to recognize the SARS-CoV-2 S-protein and modulate the renin-angiotensin system (RAS), helping to counteract the virus’s infection mechanism [209]. For the future, further studies using zebrafish could be conducted to investigate the protective role of HSA during SARS-CoV-2 infection in vivo.

### 4.3. Zebrafish Model to Study Human Infectious Diseases Caused by Fungi

Although zebrafish is a well-established model for studying bacterial and viral infections, it has not been commonly utilized to model fungal diseases. However, some studies have employed zebrafish to demonstrate the role of the innate immunity during *A. fumigatus* and *C. albicans* infections [168,169,170,210,211,212].

#### 4.3.1. *Aspergillus fumigatus*

*A. fumigatus* is an airborne pathogen that primarily threatens humans through its asexual spores, known as conidia. These tiny conidia, measuring between 2 to 3 μm, are ubiquitous, enabling them to easily reach the deep areas of the lungs when inhaled. In individuals with a healthy immune system, these inhaled conidia are typically managed effectively, posing no significant health risks. However, individuals with compromised immune systems are at risk of developing invasive aspergillosis (IA), a severe condition in which the conidia germinate into tissue-penetrating hyphae, spreading beyond the initial infection site. While defects in adaptive immunity can increase susceptibility to IA, most cases arise from weakened innate immune defenses. Mortality rates from IA can reach up to 90% in the most vulnerable populations.

Zebrafish larvae infected with *A. fumigatus* exhibit innate phagocyte populations with specific preferences for different fungal forms: macrophages rapidly phagocytose conidia and form aggregates around hyphae, whereas the neutrophil response is influenced by the presence of hyphae. Macrophage depletion renders the host larvae more susceptible to invasive diseases [170].

After germination, hyphae can be targeted by both neutrophils and macrophages, and direct contact-mediated killing by these immune cells has been observed in zebrafish larvae [170,213]. However, hyphae can develop resistance to neutrophils and antifungal treatments. Recent studies have identified the C2H2 zinc finger transcription factor A (ZfpA), which regulates *A. fumigatus* hyphal development, as a promoter of resistance to neutrophil killing and antifungal-induced stress during *A. fumigatus* infection [210]. Deletion of the *ZfpA* gene enhances fungal clearance and reduces virulence in wild-type zebrafish [210]. Interestingly, this virulence reduction is not observed in neutrophil-deficient zebrafish. Moreover, overexpression of ZfpA protects against the antifungal drug caspofungin by increasing chitin synthesis during hyphal growth. In contrast, deleting ZfpA decreases cell wall chitin and increases susceptibility to caspofungin in neutrophil-deficient zebrafish [210]. These findings provide valuable insights into fungal immune evasion and pharmacological resistance mechanisms, suggesting potential therapeutic strategies.

#### 4.3.2. *Candida albicans*

*C. albicans* is a human commensal and a clinically important fungal pathogen that grows in both yeast and hyphal forms during human infection. Although *C. albicans* can cause cutaneous and/or mucosal disease, invasive systemic infections result in the highest mortality rates among nosocomial infections [214].

To establish a disseminated infection in transparent zebrafish larvae, several routes were tested, including immersion, caudal vein injection, duct of Cuvier injection, and hindbrain ventricle injection [168]. Using the hindbrain infection route, it was found that *C. albicans* disseminated throughout the fish, with both yeast and filamentous forms reaching the tail. It was observed that *C. albicans* rapidly switches to hyphal growth but reverts to the yeast form following immune infiltration. To determine whether the immune response could account for this switch back to the yeast form, immune response cells were noninvasively visualized. This was achieved using transgenic fish expressing the enhanced green fluorescent protein (EGFP) in macrophage-like cells and endothelial tissues [215,216] as well as EGFP-expressing neutrophils [18,168]. Results obtained indicated that both macrophage-like cells and neutrophils phagocytosed yeast-form *C. albicans* and wrapped themselves around filamentous fungi [168,217].

In another study where infection was performed via immersion, the activation of the NF-κB transcriptional pathway was assessed using NF-κB transgenic zebrafish. It was observed that infection with *C. albicans* leads to NF-κB activation in epithelial cells [169]. In cases of high-level infection, the swim bladder epithelium exhibited strong NF-κB-driven EGFP fluorescence. However, in fish with a low number of yeasts in the swim bladder, there were no significant differences in NF-κB activity compared to uninfected fish. These findings suggest that at low pathogen density, an effective phagocyte response limits NF-κB activation in epithelial cells. In contrast, during high-level infection, *C. albicans* is poorly contained, leading to widespread NF-κB activation in the epithelial layer lining the swim bladder. Additionally, a strong neutrophil response was observed in the swim bladder, independent of the infection level. More neutrophils were present in both high- and low-level infections compared to uninfected controls. These results indicate that *C. albicans* infection in the swim bladder is associated with increased neutrophil recruitment, particularly during high-level infection. The robust recruitment and/or retention of neutrophils in the infected swim bladder is consistent with observations in mammalian mucosal candidiasis [169,218,219].

## 5. Conclusions

In conclusion, zebrafish turns out to be an excellent non-mammalian vertebrate model for studying infectious diseases, and specifically sensing pathways involved in the innate immune response. While zebrafish are not meant to replace other vertebrate models like mice, they offer valuable insights into microbial pathogenesis and host defense mechanisms, which could contribute to the development of innovative therapies for human infections. 

Globally, zebrafish offer numerous advantages as a model organism compared to mice, including easy imaging at single-cell resolution, large-scale WISH screens, in vivo morpholino applications, high-throughput drug screening, and the very low cost of maintaining adult zebrafish and embryos. The rapid development of genetic tools and resources for studying innate immunity in zebrafish has further driven discoveries, highlighting new technologies and approaches to uncover immune genes and their functions in health and disease. On the other hand, mammals models (e.g., murine models), which have an innate and adaptive immune system very similar to that of humans, are particularly useful for investigating the adaptive immune system and the interactions between the two systems in the context of infections caused by viruses, bacteria, and parasites [6,146,220,221].

Expanding the use of genetic screens for both host and microbes, along with chemical and toxicological screens in the zebrafish model, will enhance the value of this non-mammalian system to the broader scientific community. Indeed, zebrafish serve as an effective in vivo model system for drug screening, for assessing the mechanisms of action and toxicity of chemicals and new biotechnological products, for evaluating the effects of both traditional and emerging pollutants, and for evaluating the ecotoxicological impacts of pollutants as well as biomonitoring species in environmental risk assessments [36].

For a further expansion of the field, more advanced tools for imaging and genetic manipulation of specific immune cell types will be required. Moreover, as most of the studies using zebrafish model have focused primarily on early embryonic and larval phases, it will be intriguing to explore how the host-pathogen interactions evolve at different stages, given that the immune systems continue to mature and adapt throughout the organism life. For example, HSA is an emerging serum protein that plays a key role in innate immunity, immunomodulation, and inflammation. Indeed, HSA is internalized into leukocytes and induces significant changes in the immune cell transcriptome, specifically in genes related to CC and type I IFN responses, through interaction with endosomal TLR signaling [222]. Therefore, zebrafish in vivo studies addressing the immunomodulatory therapeutic potential of this molecule would be of great interest.
